# Silencing of DLGAP5 by siRNA Significantly Inhibits the Proliferation and Invasion of Hepatocellular Carcinoma Cells

**DOI:** 10.1371/journal.pone.0080789

**Published:** 2013-12-04

**Authors:** Weijia Liao, Weilong Liu, Qing Yuan, Xing Liu, Ying Ou, Songqing He, Shengguang Yuan, Liling Qin, Qian Chen, Kate Nong, Minghui Mei, Jian Huang

**Affiliations:** 1 Institute of Hepatobiliary Surgery, Hospital Affiliated of Guilin Medical University, Guangxi Key Laboratory of Molecular Medicine, People’s Republic of China in liver injury and repair, Hepatology Institute of Guilin Medical University, Guilin, Guangxi Zhuang Autonomous Region, China; 2 Guangdong Key Laboratory of Emerging Infectious Diseases, Shenzhen Third People's Hospital, Shenzhen, Guangdong, China; 3 Shanghai-MOST Key Laboratory for Disease and Health Genomics, National Engineering Center for Biochip at Shanghai, Shanghai, China; University of North Carolina School of Medicine, United States of America

## Abstract

**Background:**

The dysregulation of oncogenes and tumor suppressor genes plays an important role in many cancers, including hepatocellular carcinoma (HCC), which is one of the most common cancers in the world. In a previous microarray experiment, we found that DLGAP5 is overexpressed in HCCs. However, whether the up-regulation of DLGAP5 contributes to hepatocarcinogenesis remains unclear.

**Methodology/Principal Findings:**

In this study, we showed that DLGAP5 was significantly up-regulated in 76.4% (168 of 220) of the analyzed HCC specimens when compared with adjacent liver tissue. DLGAP5 overexpression was evident in 25% (22 of 88) of the HCC specimens without AFP expression, suggesting that DLGAP5 may be a novel biomarker for HCC pathogenesis. The silencing of DLGAP5 gene expression by RNA interference significantly suppressed cell growth, migration and colony formation in vitro. The expression level of DLGAP5 was also found to be related to the methylation level of its promoter in the HCC specimens.

**Conclusions/Significance:**

Taken together, these data suggest that the expression of DLGAP5 is regulated by methylation and that the up-regulation of DLGAP5 contributes to HCC tumorigenesis by promoting cell proliferation.

## Introduction

Hepatocellular carcinoma (HCC) poses a significant threat to our health due to its high incidence rate, high degree of malignancy and poor prognosis [1,2]. For nearly half a century, the prognosis of HCC has been pessimistic, despite improvements in the postoperative survival rate of HCC and the considerable progress that has been made in understanding its epidemiology, etiology, fundamental biology, diagnosis and treatment. The low recurrence-free survival (RFS) rate of 31-69% [3-5] within 5 years following surgery represents a major obstacle in improving the prognosis of HCC patients. Furthermore, the molecular mechanisms of HCC are still unclear.

The pathogenesis of HCC is a multifactorial process that involves multiple genes. Loss of tumor suppressor gene function(s), such as that of p53, and activation or overexpression of certain proto-oncogenes may all play a role in the various stages of HCC development. Specifically, the identification of oncogenes is important for HCC diagnosis, treatment and prevention as well as for the development of effective measures that would improve the outcomes of surgical treatments for HCC.

We previously searched for oncogenes in HCC by comparing the gene expression profiles of HCC and adjacent non-cancerous tissues and found that DLGAP5 is overexpressed in HCC at a high frequency [6,7]. Tsou et al [8] also reported that DLGAP5 is up-regulated in HCC. However, it remained unclear whether the up-regulation of DLGAP5 contributes to hepatocarcinogenesis. In this study, we found that the up-regulation of DLGAP5 contributes to HCC tumorigenesis by promoting cell proliferation.

## Methods

### Patients, tissue specimens and cell lines

A total of 220 pairs of HCC tissues and their adjacent non-HCC tissues were obtained from patients who underwent surgical tumor resections at the Affiliated Hospital of Guilin Medical University in China from November 2001 to April 2007. These patients were diagnosed based on clinical symptoms, serological tests, ultrasonography (US), computed tomography (CT) scans, magnetic resonance imaging (MRI) and pathological evaluations according to “Primary Liver Cancer Clinical Diagnosis and Staging Criteria” [9]. The clinicopathological characteristics for these patients, including age, gender, family history, HBsAg expression, alpha-fetoprotein (AFP) level, tumor size and number, presence of combined liver cirrhosis, history of wine-drinking, history of smoking, barcelona-clinic liver cancer (BCLC) stage, presence of portal vein tumor thrombus (PVTT), presence of distant metastasis and lymph node metastasis and incidence of postoperative recurrence, are shown in Table 1. In addition, eight specimens of normal liver tissues surrounding the hepatic hemangioma tissues were collected. All of the normal tissues were verified by pathology after the operations. In addition, 10 cases of fetal tissues were taken from educed fetuses in the Department of Obstetrics in the Affiliated Hospital of Guilin Medical University in China. All of the samples above were frozen in liquid nitrogen and placed at -80°C immediately after the surgical resections. This study was approved by the ethics committee of Hospital Affiliated of Guilin Medical University. All patients provided their written informed consent to participate in this study according to the Declaration of Helsinki. Normal liver cell lines (including LO2 and WRL68) and HCC cell lines (including Hep3B, SK-hep1, Focus, Huh7, SMMC7721, MHCC97L, MHCC97H, MHCC-LM3, MHCC-LM6, PLC, HepG2, YY8103, QGY7701, QGY7703, BEL7402, BEL7404 and BEL7405) were also used in this study. Of which Hep3B, SK-hep1, Focus, Huh7, SMMC7721, PLC, HepG2, YY8103, QGY7701, QGY7703, BEL7402, BEL7404 and BEL7405 were derived from commercial source (Institute of chemistry and cell biology at shanghai). And MHCC97L, MHCC97H, MHCC-LM3 and MHCC-LM6 were derived from published references 10,11, kindly provided by professor Yinkun Liu from Liver Cancer Institute affiliated Zhongshan Hospital at shanghai. 

**Table 1:** Summary of Analyses of DLGAP5 gene in 220 HCC tissues.

**Clinical characterization**	**Clinical group**	**No. of patients**	DLGAP5 High*(%)	DLGAP5 Low*(%)	**χ^2^**	**p value**
Age (years)	<50	118	91 (54.17)	27 (51.92)	0.08	0.777
	≥50	102	77 (45.83)	25 (48.08)		
Gender	Male	187	147 (87.50)	40 (76.92)	3.484	0.062
	Female	33	21 (12.50)	12 (23.080		
Family history	No	187	144 (85.71)	43 (82.69)	0.284	0.594
	Yes	33	24 (14.29)	9 (17.31)		
HBsAg	Negative	39	28 (16.67)	11 (21.16)	0.548	0.459
	Positive	181	140 (83.33)	41 (78.84)		
α-fetoprotein (μg/l)	<100	79	54 (32.14)	25 (48.08)	4.381	0.036
	≥100	141	114 (67.86)	27 (51.92)		
Median size (cm)	<5	49	38 (22.62)	11 (21.15)	0.049	0.824
	≥5	171	130 (77.38)	41 (78.85)		
Cirrhosis	No	21	14 (8.33)	7 (13.46)	1.209	0.271
	Yes	199	154 (91.67)	45 (86.54)		
Tumor number	Single	149	113 (67.26)	36 (69.23)	0.07	0.791
	Multiple	71	55 (32.74)	16 (30.77)		
Wine-drinking	No	103	78 (46.43)	25 (48.08)	0.043	0.835
	Yes	117	90 (53.57)	27 (51.92)		
Smoking	No	105	77 (45.83)	28 (53.84)	1.022	0.312
	Yes	115	91 (54.17)	24 (46.16)		
BCLC stage	0	7	3 (1.78)	4 (7.69)	1.834	0.176
	A	96	72 (42.86)	24 (46.15)		
	B	43	33 (19.64)	10 (19.23)		
	C	74	60 (35.71)	14 (26.92)		
Portal vein tumor thrombus	No	167	121 (72.02)	46 (88.46)	5.867	0.015
	Yes	53	47 (27.98)	6 (11.54)		
Distant metastasis	No	195	149 (88.69)	46 (88.46)	0.002	0.964
	Yes	25	19 (11.31)	6 (11.54)		
Lymph node metastasis	No	210	160 (95.24)	50 (96.15)	0.077	0.782
	Yes	10	8 (4.76)	2 (3.85)		
Recurrence	No	143	103 (61.31)	40 (76.92)	4.255	0.039
	Yes	77	65 (38.69)	12 (23.08)		

* DLGAP5 High: 2**^*−*^**ΔΔ^CT^>1; DLGAP5 Low: 2**^*−*^**ΔΔ^CT^≤1.

We followed up on all of the HCC patients after surgery by measuring their serum alpha-fetoprotein (AFP) levels and performing ultrasonography (US) every 2 months and chest radiography every 6 months during the first 2 postoperative years and at 3- to 6-month intervals thereafter. A CT scan or MRI was performed if recurrence was suspected due to an abnormal AFP level or ultrasonograph. Disease-free survival was measured from the day after surgery to the date of recurrence, metastasis, death or last follow-up. Among the 220 HCC patients, the average disease-free survival time was 34.4 months (median, 21.0 months; range, 2.0 to 72.0 months). 

### RNA Extraction and cDNA Synthesis

Total RNA was extracted from HCC cell lines or frozen tissue samples that were pulverized under liquid nitrogen using TRIzol (Invitrogen, Carlsbad, CA, USA). To reduce the risk of genomic DNA contamination, 1-2 μg RNA was incubated with 2 U DNase I (Invitrogen, Carlsbad, CA, USA), 1 μl DNase buffer and 0.4 μl RNase Out for 15 min at room temperature. The RNA concentration was determined by spectrophotometry, and the total RNA integrity was examined by visualization of the 28S and 18S ribosomal RNAs on a 1.2% agarose gel. First-strand cDNA was synthesized using the Prime Script RT Reagent Kit (TaKaRa, Otsu, Japan) according to the manufacturer’s instructions.

### Semi-quantitative RT-PCR

Reverse transcription (RT) was performed with 2 μg total RNA that was treated with RNase-free DNase I. Semi-quantitative RT-PCR was then performed using the following primers for DLGAP5: 5′-GGAAGACCTGCCAAAAATGTAG-3′ (forward) and 5′-TGTGCCAAAATTTCTTTTGTTG-3′ (reverse). The length of the amplified fragment was 350 bp. β-Actin served as the internal reference. The primers for β-actin are as follows: 5′-TCACCCACACTGTGCCCATCTACGA-3′ (forward) and 5′-CAGCGGAACCGCTCATTGCCAATGG-3′ (reverse). The length of the β-actin amplicon was 295 bp. The PCR products were separated on 2% agarose gels containing ethidium bromide. The PCR reaction was performed in a volume of 20 μl using the TaKaRa PCR Kit. The reactions were performed at 94°C for 5 min followed by 35 cycles (for DLGAP5) or 25 cycles (for β-actin) of 94°C for 30 sec, 55°C for 30 sec and 72°C for 30 sec, and a final extension at 70°C for 5 min. The PCR products were brought to 4°C at the end of the reaction, and they were then separated on 2% agarose gels containing ethidium bromide.

### Quantitative real-time PCR

Quantitative real-time PCR (qRT-PCR) was performed using SYBR Premix Ex Taq according to the manufacturer’s instructions. The primer sequences for DLGAP5 were 5′-CATCTGGAATGTCCAATTCAAG-3′ (forward) and 5′-ATGAAGGTCGAATT GCTCAG-3′ (reverse), and the length of the amplicon was 129 bp. The primer sequences of the internal reference gene β-actin were 5′-GACAGGATG CAGAAGGAGATTACT-3′ (forward) and 5′-TGATCCACA TCTGCTGGAAGGT-3′ (reverse), and the length of the amplicon was 142 bp. Real-time polymerase chain reaction amplification and data analysis were performed using the ABI Prism 7500 Sequence Detector System (Applied Biosystems, Foster City, CA, USA). Each cDNA sample was mixed with 15 μl SYBR Green PCR Master Mix (Applied Biosystems, Foster City, CA, USA). The PCR protocol consisted of an initial denaturation step at 95°C for 10 min, followed by 40 cycles of denaturation at 95°C for 30 sec, annealing at 55°C for 30 sec and extension at 72°C for 30 sec, and fluorescence acquisition at 72°C. The mean Ct value for β-actin was subtracted from the mean Ct value for DLGAP5 in each sample using the following formula: DLGAP5 ΔCt = (mean DLGAP5 Ct – mean β-actin Ct). The fold change (2^–DLGAP5ΔΔCt^) of the DLGAP5 expression level relative to the β-actin expression level was calculated for each sample [12].

### Immunohistochemistry (IHC)

Formalin-fixed, paraffin embedded tissue blocks and the corresponding hematoxylin-eosin (H&E)-stained slides were overlaid for tissue microarray sampling. HCC and non-HCC tissue array slides (12 cases/24 cores) were reviewed by two histopathologists, and representative tumor areas that were free from necrotic and hemorrhagic materials were pre-marked in the paraffin blocks.

The tissue microarray slides were deparaffinized in xylene, rehydrated in a graded ethanol series, antigen-retrieved by pressure cooking for 3 min in ethylenediamine tetraacetic acid (EDTA) buffer (pH 8.0) and washed in phosphate-buffered saline (PBS). The slides were then immersed in 3% hydrogen peroxide for 20 min to block endogenous peroxidase activity and pre-incubated with 10% normal goat serum at room temperature for 30 min to reduce nonspecific binding. Subsequently, the slides were incubated with a rabbit polyclonal anti-DLGAP5 antibody (Santa Cruz Biotechnology, Inc., 1:200 dilution) overnight in a moist chamber at 4°C, washed in PBS, incubated with a biotinylated goat anti-rabbit antibody for 1 hour at room temperature and stained with 3,3-diaminobenzidine tetrahydrochloride (DAB). Finally, the sections were counterstained with Mayer’s hematoxylin, dehydrated and mounted. A negative control was prepared by replacing the primary antibody with normal rabbit or mouse IgG. A semi-quantitative IHC detection method was used to determine the DLGAP5 protein levels, and the stained tissue sections were assessed on a four-point scale based on positive cell counts that were graded from 0 to 3 (0, no positive cells; 1, <25% positive cells; 2, 25-50% positive cells; 3, >50% positive cells). The slides were independently reviewed under light microscopy by two pathologists.

### Western blotting

Total cellular protein was extracted using lysis buffer [50 mM Tris-HCl (pH 7.4), 1 mM EDTA, 250 mM NaCl, 1% Triton X-100, 5 mM MgCl_2_, 2 mM Na_3_VO_4_, and 1× Complete™ protease inhibitor]. Protein concentration was measured using Bio-Rad protein assay. Equal amount of protein was separated using 10% SDS-PAGE and transferred to nitrocellulose membranes (Amersham, GE Healthcare, New Jersey, USA). The membranes were blocked with 5% skimmed milk in PBS buffer and incubated overnight with primary antibody (Santa Cruz Biotechnology, Inc., 1:500 dilution) at 4°C. β-actin was used as internal positive control. After applying a secondary antibody followed by horseradish peroxidase conjugated, immunodetection was performed with enhanced chemiluminescence, detected on X-ray films (Fuji films).

### RNA interference and cell growth assay

Three siRNAs against DLGAP5 were designed on the Whitehead Institute Web Server (http://jura.wi.mit.edu/bioc/siRNAext/) and chemically synthesized (Shanghai GenePharma Co.) to target different coding regions of the gene. The siRNA sequences are as follows: siRNA-1 (5′-GGUGGCAAGUCAAUAAUAATT-3′ and 5′-UUAUUAUUGACUUGCCACCTT-3′), siRNA-2 (5′-GUGCCAUAUUUCAGAA AUATT-3′ and 5′-UAUUUCUGAAAUAUGGCACTT-3′), and siRNA-3 (5′-GGGCAUUCCACAACAAACUTT-3′, and 5′-AGUUUGUUGUGGAAUGC CCTT-3′). In addition, siRNA-NC (5′-GAGUUAAAGUCAAAGUGACTT-3′ and 5′-GUCACUUUGACUUUAACUCTT-3′) was also synthesized. All of the siRNAs were transfected into HCC cell lines, and cell growth was monitored. For siRNA transfection, 3 × 10^3^ HCC cells per well were seeded in 96-well plates. When the cells reached 30%-50% confluence, they were transfected with the synthetic siRNAs at a final concentration of 50 nM using Lipofectamine 2000 Transfection Reagent (Invitrogen) according to the manufacturer’s instructions. The cells were then cultured for seven days. Cell viability was measured using the CCK-8 according to the manufacturer’s instructions. Briefly, 10 μl CCK-8 solutions were added to each well of the plate, and the plate was incubated at 37°C for 1 hour. The absorbance was measured at 450 nm to assess cell viability. All experiments were independently repeated at least three times.

### Wound-healing assay

Cells were grown to 80%-90% confluence in 60-mm plates and then transfected with shRNA-2 to knock down DLGAP5 expression. A wound was made by dragging a plastic pipette tip across the cell culture surface 48 hours after transfection. The cells were then incubated at 37°C, and phase contrast images of the wound were recorded at 0, 12, 24 and 36 hours. Three separate experiments were performed. Cells that were transfected with shRNA-NC served as the control.

### Cell invasion assay

Cell invasion assays were performed using 24-well Transwells (8-μm pore size; BD Biosciences) that were coated with Matrigel (Falcon 354480; BD Biosciences). HCC cells were starved overnight in serum-free medium, trypsinized and washed three times in DMEM containing 1% FBS. A total of 1 × 10^5^ cells were then resuspended in 500 μl DMEM containing 1% FBS and added to the upper chamber, while 750 μl DMEM containing 10% FBS and 10 μg/ml fibronectin (BD Biosciences, San Jose, CA, USA) was placed in the lower chamber. For the control, medium containing 1% FBS was added to the lower chamber. After 48 hours of incubation, the Matrigel and the cells remaining in the upper chamber were removed by cotton swabs. The cells on the lower surface of the membrane were fixed in 4% paraformaldehyde and stained with 0.5% crystal violet. The cells in at least six random microscopic fields (magnification, ×100) were counted and photographed. All experiments were performed in duplicate and repeated three times.

### Cell adhesion assay

Fibronectin by PBS dilutions after coated 96-well plates each well 50μl, control cell culture put in 50μl medium, and incubated overnight at 4°C. The next day washed with PBS once, add 1% BSA for blocking, each well 150μl, at 37°C for 1 hour and then washed with pre-warmed serum-free medium can be used for cell inoculation once. 3 × 10^3^ growth arrest prior starvation phase HCC cells per well were seeded in 96-well plates each well 100μl. Cell adhesion ability was measured using the CCK-8 according to the manufacturer’s instructions. The absorbance was measured at 450 nm to assess cell adhesion ability. All experiments were independently repeated at least three times.

### Bisulfite DNA sequencing

Genomic DNA was extracted from the tissue samples using the DNeasy Tissue Kit (Qiagen) according to the manufacturer’s instructions. Bisulfite treatment of the genomic DNA was performed using the EpiTect Bisulfite Kit (Qiagen) according to the manufacturer’s instructions. For bisulfite DNA sequencing, the CpG island was amplified using the primers 5′-AGTGGAAGGGGTGGGATT-3′ (forward) and 5′-TCCAACACCCACCTACCTAA-3′ (reverse), which are specific to the DLGAP5 gene promoter. The PCR products were purified and subcloned into the pMD18-T vector (TaKaRa). Random colonies were selected for sequencing.

### Statistical analysis

The statistical analyses were performed using SPSS13.0 software. Survival curves were generated using the Kaplan-Meier method and checked for statistical significance with the log-rank test. Qualitative variables were compared with the Pearson’s chi-square test, and quantitative variables were analyzed by the independent t test. Chi-Square Test was used for correlation analysis. The statistical significance of the differences between mean values was defined as *P* <0.05.

## Results

### DLGAP5 mRNA is frequently up-regulated in HCC

**Figure 1:**
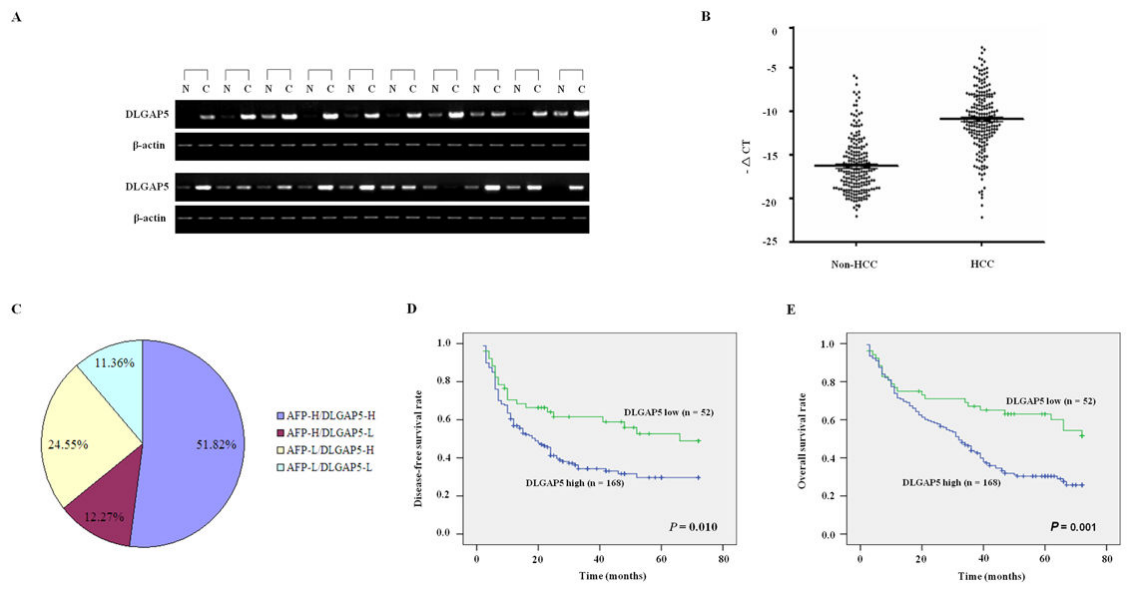
mRNA expression pattern of DLGAP5 in HCC specimens. (A) Representative results of the semi-quantitative RT-PCR analysis of DLGAP5 in 20 pairs of HCC (C) and adjacent non-cancerous liver tissues (N). β-Actin was used as an internal control. Each PCR was performed for 35 cycles, and the PCR products were visualized by electrophoresis on 2% agarose gels. (B) Real-time RT-PCR analysis of DLGAP5 in 220 paired HCC and adjacent non-cancerous liver tissues. For each sample, the relative mRNA level of DLGAP5 was normalized to the level of β-actin. The line within each box represents the median –ΔCt value; the upper and lower edges of each box represent the 75th and 25th percentile, respectively; the upper and lower bars indicate the highest and lowest values determined, respectively. (C) The distribution of the DLGAP5 and AFP expression levels in the 220 HCC specimens. The numbers indicate the percentages of DLGAP5- and/or AFP-positive HCC specimens, as detected by real-time RT-PCR. (D)/(E) Disease-free survival rate (D) and Overall survival rate (E) of patients with HCC. High expression of DLGAP5 mRNA was significantly associated with worse prognosis (*P*=0.010). DLGAP5 high: 2^−ΔΔCT^>1 (or –ΔΔCT>0); DLGAP5 low: 2^−ΔΔCT^≤1 (or −ΔΔCT≤0).

We found that DLGAP5 was significantly up-regulated in 18 of the 20 analyzed HCC specimens (90%) compared with their adjacent non-cancerous liver tissues using a semi-quantitative RT-PCR assay (Figure 1A). Considering the limitations of the RT-PCR method, the mRNA level of DLGAP5 was further evaluated in 220 informative cases by real-time RT-PCR. After normalizing to β-actin, 168 of the 220 HCC cases (76.4%) showed a ≥2-fold increase of the DLGAP5 mRNA compared with the corresponding non-cancerous livers (*P*<0.001, Figure 1B). This finding further confirmed that DLGAP5 expression is significantly increased in HCC relative to non-cancerous liver tissues.

Despite this finding, the up-regulation of DLGAP5 was not statistically correlated with gender, age (≥50 or <50), tumor size (≥5 or <5 cm), family history, hepatitis B surface antigen (HBsAg) expression, presence of liver cirrhosis, history wine-drinking, history of smoking, BCLC stage, or presence of distant metastasis or lymph node metastasis (*P*>0.05, Table 1). However, the up-regulation of DLGAP5 positively correlated (*P*<0.05) with the level of alpha-fetoprotein (AFP) (*P*=0.036), the presence of portal vein tumor thrombus (PVTT) (*P*=0.015) and postoperative recurrence (χ^2^=4.255, *P*=0.039, Table 1). 

Interestingly, the expression of DLGAP5 and AFP did not completely overlap in the 220 HCC specimens, as examined by real-time RT-PCR. DLGAP5 were both overexpressed in 114 of the 220 HCC specimens (51.82%) with AFP positive. Moreover, DLGAP5 was not significantly expressed in only 27 (12.27%) cases that were AFP positive. Surprisingly, overexpression of DLGAP5 but not AFP was evident in 24.55% (54 of 220) of the HCC specimens (Figure 1C), suggesting that DLGAP5 might be a novel biomarker for HCC and that the simultaneous detection of AFP and DLGAP5 could improve the sensitivity of histological diagnosis to 88.64%.

We then analyzed the disease-free survival rates and overall survival rates to assess prognostic significance of DLGAP5 mRNA expression. Five-year disease-free and overall survival rates of the 220 patients with HCC were 40.1 and 45.2%, respectively. Patients who had higher DLGAP5 expression levels had shorter 5-year survival rates than patients who had lower DLGAP5 expression levels (disease-free survival rate, 30.4% *vs* 55.8%; *P*=0.002; overall survival rate, 31.6% *vs* 62.9%; *P*=0.001), when assessed by Kaplan-Meier curves (Figure 1D and 1E).

### DLGAP5 Overexpression Is Closely Correlated with Intrahepatic Metastasis

**Figure 2:**
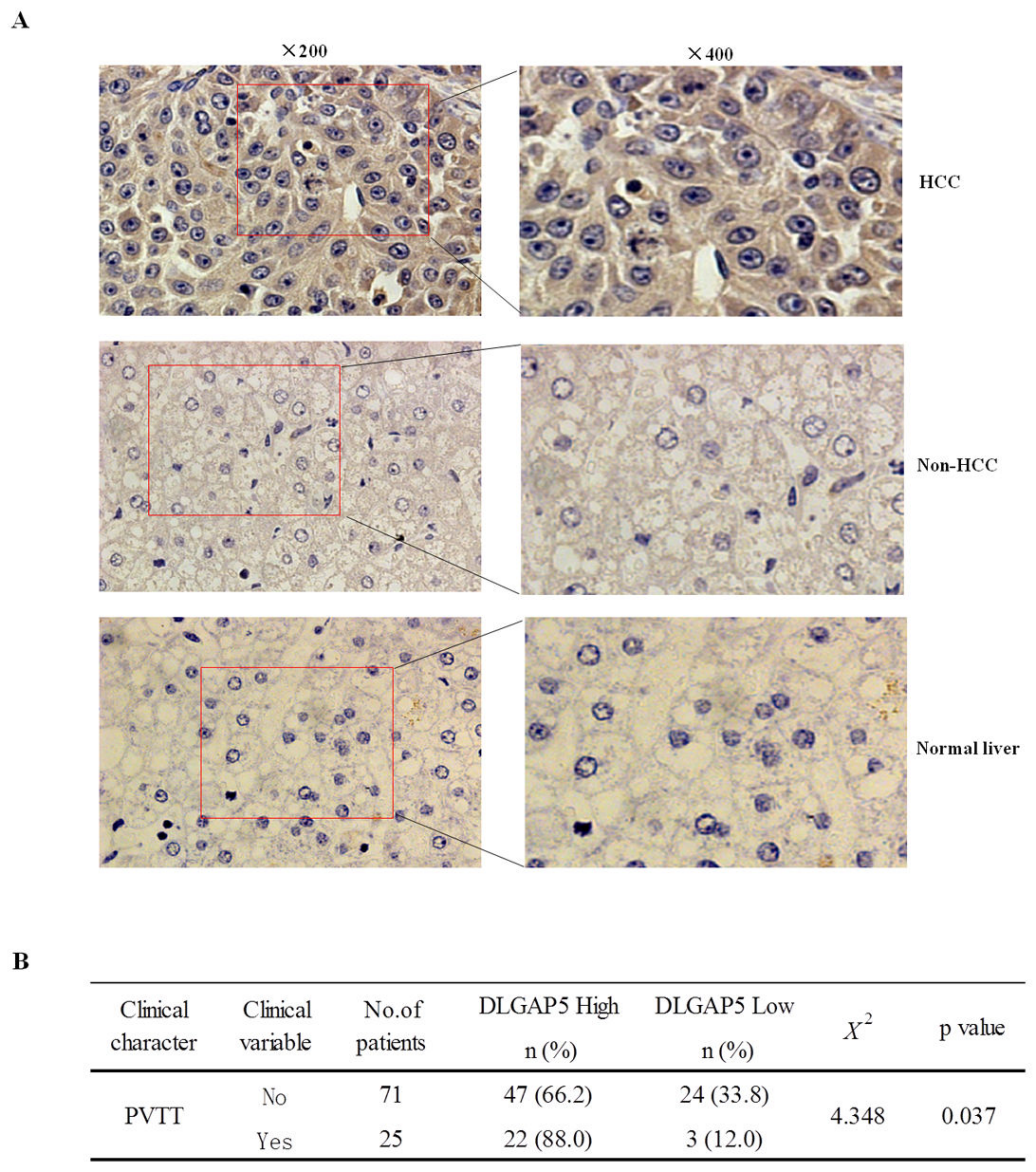
Protein expression pattern of DLGAP5 in HCC specimens. (A) Representative immunohistochemical DLGAP5 staining of a HCC specimen and its corresponding non-cancerous tissue from a tissue array containing 96 pairs of HCC specimens. The nuclei were counterstained with hematoxylin. Original magnification: The left (×200) and right side (×400) (normal, non-HCC and HCC). (B) Statistical analysis was performed using the chi-square test to compare the relative levels of DLGAP5 between HCC with PVTT and HCC without PVTT (*P*=0.037).

To further validate the up-regulation of DLGAP5 at the protein level, immunohistochemical staining was performed on an additional 96 pairs of HCC and corresponding adjacent non-cancerous liver tissues using an in-house custom tissue array, and the staining intensity was scored on a scale of 0 to 3. Interestingly, DLGAP5 was found to be increased in 69 of the 96 HCC specimens (71.87%) compared with their adjacent non-cancerous liver tissues (*P*<0.05). Furthermore, only 10 of the 96 non-HCC tissues (10.4%) had positive DLGAP5 staining. The staining patterns also showed that DLGAP5 was mostly anchored in the cytoplasm and the extracellular matrix (ECM) (Figure 2A). 

Surprisingly, the DLGAP5 levels were significantly higher in the HCC specimens with PVTT than in those without PVTT (*P*<0.05; Figure 2B), suggesting that DLGAP5 overexpression is associated with cellular invasion, venous permeation and perhaps even metastasis in HCC.

**Figure 3:**
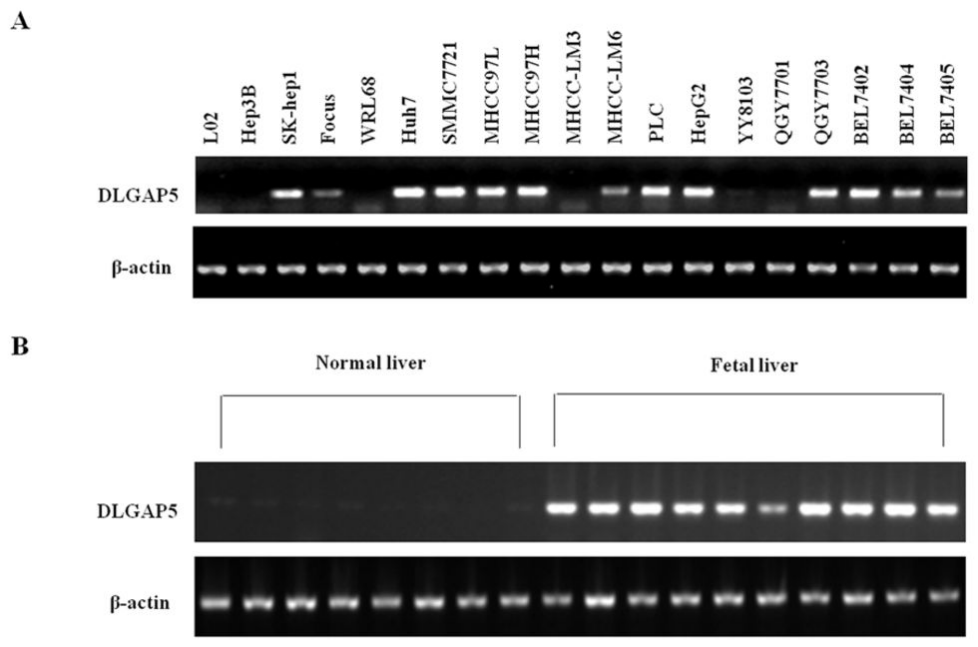
mRNA expression pattern of DLGAP5 in HCC cell lines, normal livers and fetal livers. (A and B) Results of the semi-quantitative RT-PCR analysis of DLGAP5 in 19 liver cancer cell lines (A) and in 8 normal liver tissues and 10 fetal liver tissues (B). β-Actin was used as an internal control. All PCR products were visualized by electrophoresis on 2% agarose gels.

We also evaluated the expression level of DLGAP5 in various HCC cell lines by RT-PCR and found that DLGAP5 was significantly expressed in SK-hep1, Huh7, SMMC7721, MHCC97L, MHCC97H, PLC, HepG2, QGY7703, BEL7402 and BEL7404 cells but not in the normal liver cell lines LO2 and WRL68 (Figure 3A). In addition, we found no positive staining for DLGAP5 in normal liver tissues, in contrast to its strong expression in normal fetal liver tissues (Figure 3B).

### Depletion of endogenous DLGAP5 through RNA interference suppresses HCC cell line growth

**Figure 4:**
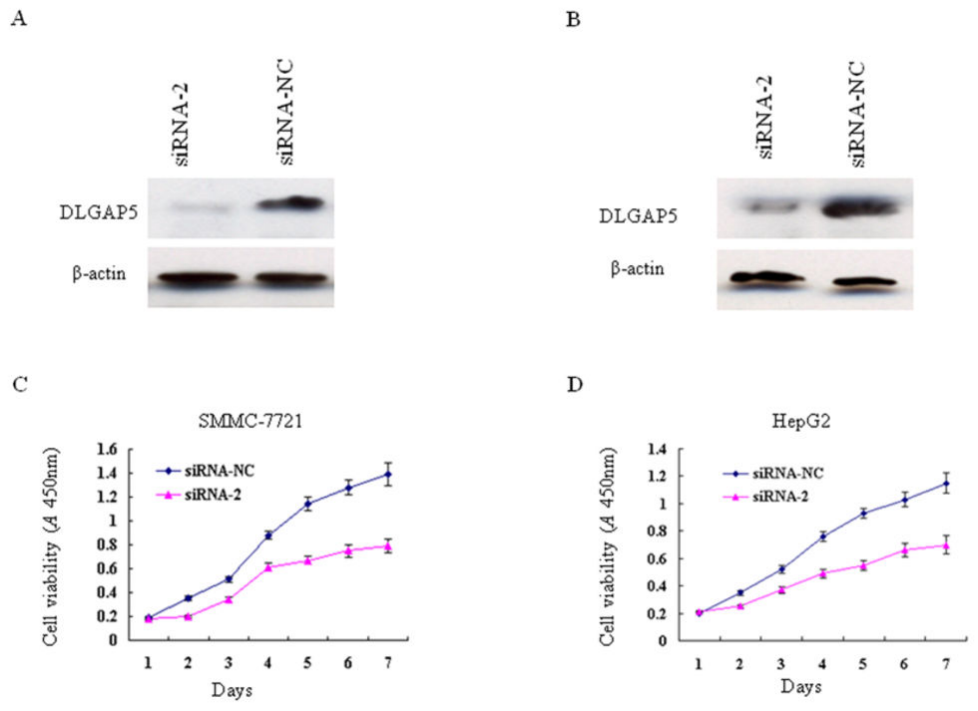
Effect of DLGAP5 silencing on HCC cell growth. (A and B) western blot confirmation of DLGAP5 knockdown in SMMC-7721 (A) and HepG2 (B) cells via transient transfection with siRNA2. siRNA-NC was used as the control. (C and D) The growth curves of the SMMC-7721 (C) and HepG2 (D) cells after DLGAP5 knockdown by siRNA2 were plotted based on the CCK-8 assay. siRNA-NC served as a control. The experiments were repeated at least three times, and the data points represent the average values of triplicate wells, with standard deviations (SDs) included for each mean value.

To further evaluate the contribution of DLGAP5 to HCC tumorigenesis, we designed three DLGAP-specific siRNAs (siRNA1, 2 and 3) for gene knockdown experiments. To test the efficacy of these siRNAs, they were transiently transfected into SMMC-7721 cells. siRNA-NC was used as a negative control. We found that siRNA2 significantly knocked down endogenous DLGAP5 expression compared with siRNA-NC (Figure S1). siRNA2 was then used for cell growth analysis after DLGAP5 knockdown in various HCC cell lines, including SMMC-7721 and HepG2. Our data showed that the transient transfection of siRNA2 suppressed the growth of the SMMC-7721 and HepG2 cells compared with siRNA-NC (*P*<0.05, Figure 4). These data further support the hypothesis that the frequent up-regulation of DLGAP5 contributes to hepatocarcinogenesis by promoting the growth of the existing HCC cells.

### DLGAP5 promotes cell migration, invasion and adhesive ratio

**Figure 5:**
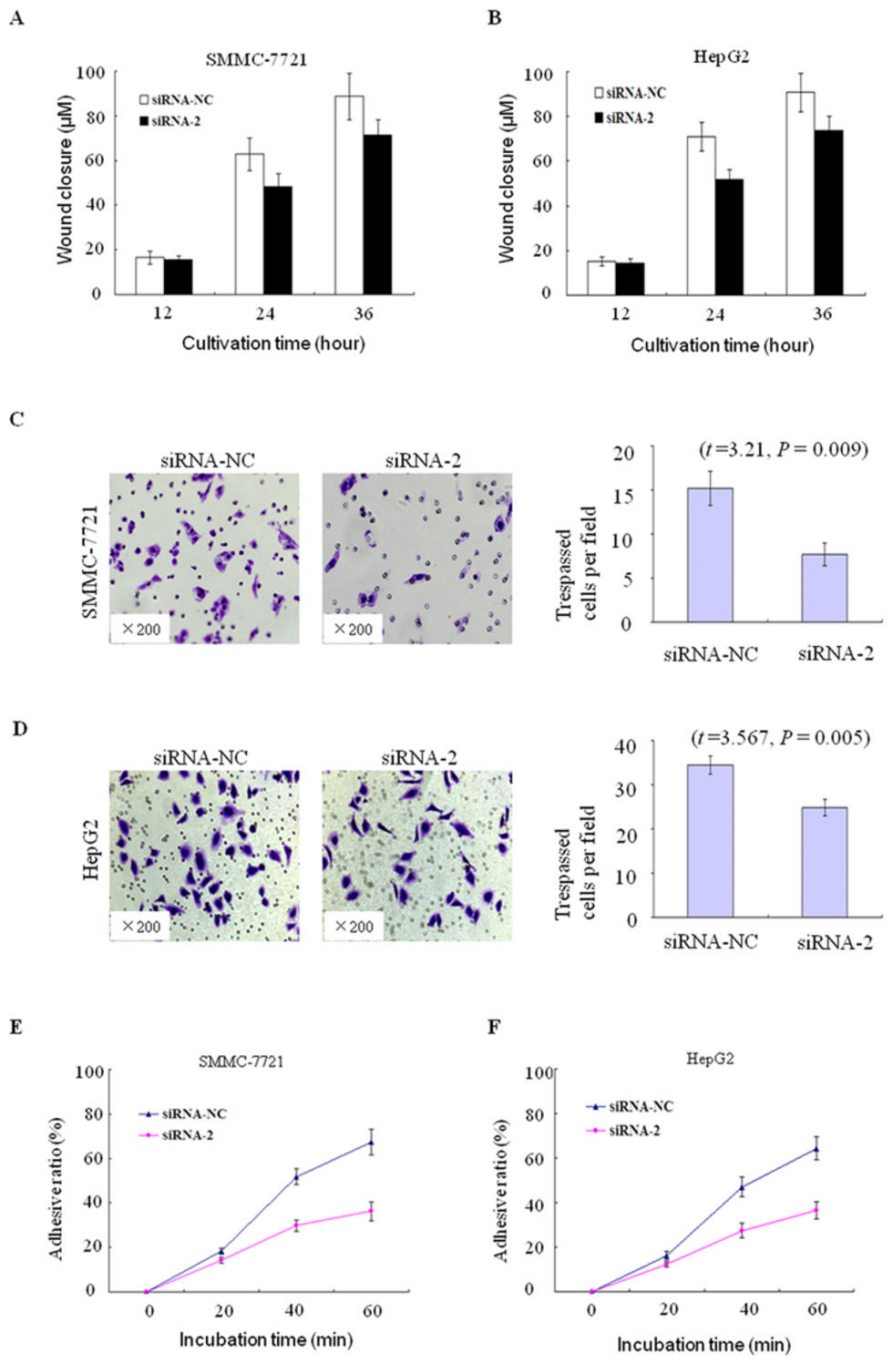
Effect of DLGAP5 on HCC cell migration, invasion and adhesive ratio. (A and B) Wound closure of SMMC-7721 (A) and HepG2 (B) cells that were transfected with siRNA2 in a wound-healing experiment, with siRNA-NC serving as a control. (C and D) Invasion of SMMC-7721 (C) and HepG2 (D) cells that were transfected with siRNA2 in a Matrigel assay, with siRNA-NC serving as a control. The cell numbers represent the mean values per field (from at least five fields) from three independent experiments (right panel) (mean ± SD). (E and F) Effect of DLGAP5 gene on SMMC-7721 and HepG2 cell adhesive ratio using RNAi.

The aforementioned tissue array data showed that the up-regulation of DLGAP5 may be closely associated with cellular invasion, venous permeation and intrahepatic metastasis in HCC (Figure 2B). We therefore performed wound-healing and Matrigel assays to assess the effects of DLGAP5 knockdown by RNAi on cell migration and invasion. The wound-healing assay revealed that cell migration was inhibited as a result of DLGAP5 knockdown in SMMC-7721 and HepG2 cells (Figure 5A and 5B, and Figure S2). In comparison with the cells that were transfected with siRNA-NC, the cells of both cell lines that were transiently transfected siRNA2 also showed a significant inhibition of cell invasion through a Matrigel barrier when fibronectin was used as an attractant (*P*<0.01; Figure 5C and 5D). Furthermore, we performed the cell adhesion assay to explore the effect of DLGAP5 gene on the ability of cell adhesion of SMMC-7721 and HepG2 cells. The results showed that the adhesion ability was significantly inhibited in SMMC-7721 (Figure 5E) and HepG2 (Figure 5F) cells transfected by siRNA2 compared with those cells transfected by siRNA-NC. Take together these results suggest that DLGAP5 has a novel role in HCC metastasis by promoting cell invasion.

### DNA methylation status of the DLGAP5 promoter is reduced in HCC tissues

To address whether genetic alterations contribute to the dysregulation of DLGAP5 in HCCs, we used a custom-designed Multiplex AccuCopy Kit (Genesky Biotechnologies Inc., Shanghai, China) to detect DNA copy number changes in HCC tissues compared with non-HCC tissues based on the previously described multiplex fluorescence competitive PCR principle. The reference genome sequences were obtained from the UCSC Genome Browser (build hg19; http://genome.ucsc.edu). Three reference genes (POP1, RPP14 and TBX15) were used for normalization [13]. No significant differences were identified between the two groups (data not shown), suggesting that genetic events are not critical for the up-regulation of DLGAP5 in HCC.

**Figure 6:**
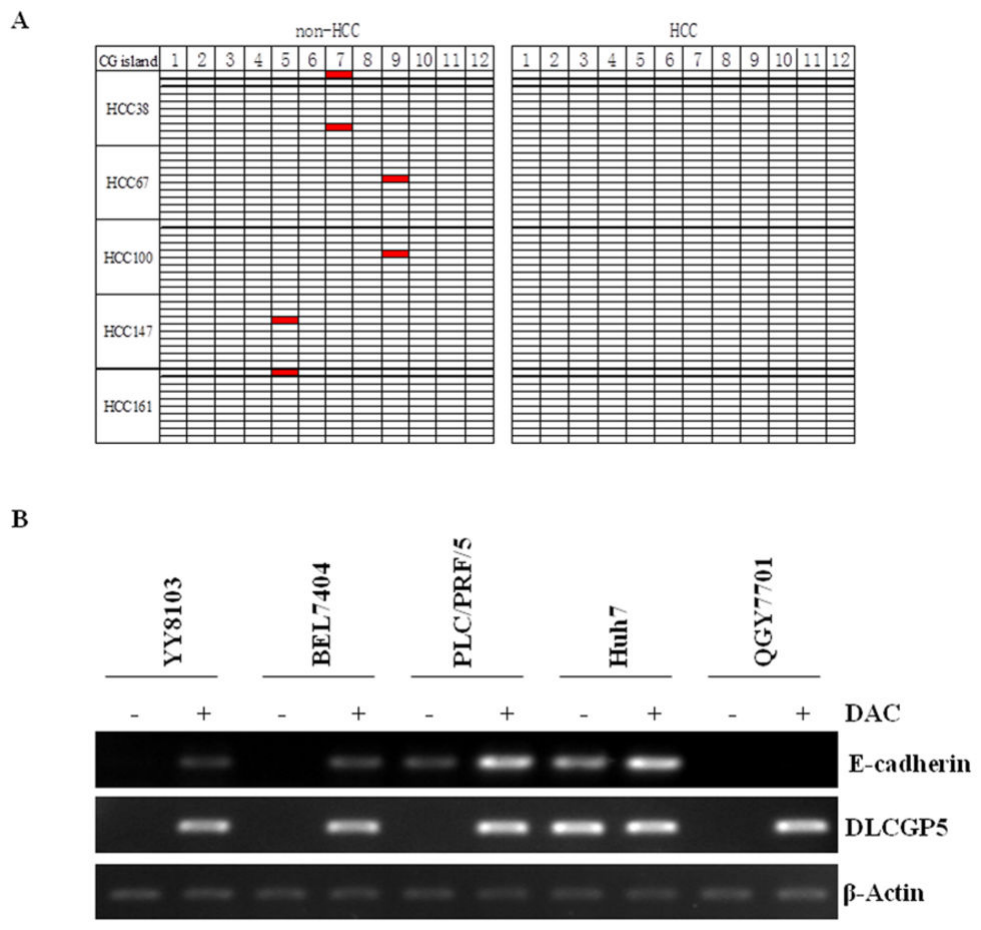
The DNA methylation status of DLGAP5 promoter in HCCs. (A) Results from the bisulfite sequencing analysis of DLGAP5 promoter CpG methylation in five pairs of HCC and non-HCC specimens. Each box indicates a CpG dinucleotide within the CpG island in the promoter. (B) YY8103, BEL7404, PLC/PRF/5, Huh7 and QGY7701 cells were treated with 5-aza-2'-deoxycytidine (DAC, work concentration: 2.0uM). The expression of DLGAP5 was then evaluated by RT-PCR. Untreated HCC cells were employed as control. E-cadherin, a well-known hypermethylation-silenced genes in HCC, was used for positive control.

In light of this finding, we next determined whether epigenetic mechanisms may be involved in the dysregulation of DLGAP5. We conducted bisulfite DNA sequencing to characterize the methylation status of the DLGAP5 promoter in five pairs of HCC and non-HCC specimens that showed DLGAP5 overexpression in HCCs compared with non-cancerous tissues. The sequencing data revealed that DLGAP5 promoter methylation was significantly reduced in all five (100%) HCC specimens compared with the paired non-cancerous liver tissues (Figure 6A). To confirm the findings, we analyzed the expression of DLGAP5 in some HCC cell lines including YY8103, BEL7404, PLC/PRF/5, QGY7701 and Huh7 treated by 5'azacytidine (DAC) compared with no drug treatment. The results showed that DLGAP5 gene can be re-expressed in YY8103, BEL7404, PLC/PRF/5 and QGY7701 HCC cell lines with drug treatment compared to those cell lines without drug treatment (Figure 6B). However, we did not observe re-expression of DLGAP5 gene in Huh7 cell line. The potential reason may be related to higher expression level of DLGAP5 gene in Huh7 cell line (Figure 3A). The current findings suggested that the upregulation of DLGAP5 in HCC is correlated with the methylation levels of promoter of DLGAP5 gene.

## Discussion

Hepatocellular carcinoma is the main type of primary liver cancer and also one of the most malignant tumors. At present, the pathogenesis of liver cancer is not entirely understood. It has been shown that the inactivation of tumor suppressor genes and the activation of oncogenes both play significant roles in carcinogenesis, which can be caused by both genetic and epigenetic aberrances. The identification of oncogenes is especially important in the search for novel biomarkers and drug targets for liver cancer. Some studies have reported that abnormal gene expression, such as that of CD151 [14], CD44 [15], CD44s [16], CK-19, MMP, OPN [17], KLF8 [18], TGF-beta [19] and HRP-3 [20], is closely associated with the development and progression of liver cancer.

Notably, DLGAP5 contains a guanylate-kinase-associated protein (GKAP) domain that is conserved among various species, including *Homo sapiens, Pan troglodytes, Macaca mulatta, Canis lupus, Bos taurus, Mus musculus, Rattus norvegicus, Gallus gallus* and *Danio rerio*. This domain is also found in many eukaryotic signaling proteins, suggesting that DLGAP5 may have important biological functions as a signaling molecule. This hypothesis is supported by our results showing that DLGAP5 is localized to the cytoplasm. In addition, Kuo et al. found that DLGAP5 knockdown inhibits the proliferation of hepatocellular carcinoma cells via the down-regulation of gankyrin and the accumulation of p53 [21].

In this study, we found that DLGAP5 was significantly up-regulated in 76.4% of the analyzed HCC specimens compared with their paired non-HCC liver tissues. Tsou et al. previously used quantitative RT-PCR to examine the gene expression pattern of DLGAP5 in 16 types of human tissues and found high expression in the testis, bone marrow, colon and placenta, while strong expression of DLGAP5 transcript in fetal liver. In contrast, no expression of DLGAP5 was found in normal adult liver tissue (Figure 3B) [8]. Therefore, DLGAP5 may likely contribute to hepatocarcinogenesis as a new cancer-testis (CT)-related gene that is involved in the de-differentiation of hepatocytes to an embryonic state.

Interestingly, the up-regulation of DLGAP5 expression in HCCs was not statistically correlated with patient gender, age, tumor size, family history, HBsAg expression, liver cirrhosis, history of wine-drinking, history of smoking, BCLC stage, or development of distant metastasis or lymph node metastasis (*P*>0.05). However, the up-regulation of DLGAP5 positively correlated with the level of AFP (*P*<0.05). This result suggests that the simultaneous detection of AFP and DLGAP5 could improve the positive ratio of histological diagnosis up to 88.64%. Similarly, Fragoso et al. reported that the combined expression of BUB1B, DLGAP5 and PINK1 may serve as an outcome predictor in adult adrenocortical tumors (ACTs) [22].

The mechanisms of DLGAP5 regulation should be further investigated. It is known that the abnormal methylation status of CpG islands in gene promoters is often linked to the inactivation of tumor suppressor genes and the activation of oncogenes in cancer [23,24]. Our data showed that the expression level of DLGAP5 correlated with the methylation level of the DLGAP5 promoter in the HCC specimens, suggesting that the expression of DLGAP5 was regulated by promoter CpG methylation.

To understand the biological function of DLGAP5 in liver cancer, we knocked down DLGAP5 gene expression by RNA interference and observed a significant suppression of cell growth and colony formation. This finding strongly supports the data from Kuo et al. and Zhao et al., which showed that DLGAP5 is a potential oncogene that promotes the proliferation of HCC cells [21,25]. 

In this study, we also found that the DLGAP5 level was significantly higher in the HCC specimens with PVTT than in those without PVTT (Figure 2B), suggesting that DLGAP5 overexpression is associated with cellular invasion, venous permeation and perhaps even metastasis in HCC. To validate this hypothesis, we performed wound-healing, Matrigel assays and cell adhesive assay to assess the effects of DLGAP5 silencing by RNAi on cell migration and invasion. Our results suggest that DLGAP5 strongly promotes the invasion of HCC cells.

Taken together, our data show that DLGAP5 plays an essential role in HCC pathogenesis. DLGAP5 is a potential oncogene that can serve as a new biomarker for HCC diagnosis or a candidate target in HCC therapy. Our results also show that epigenetic mechanisms, rather than genetic events, may play an important role in the regulation of DLGAP5 expression in HCC. Moreover, we reveal that DLGAP5 promotes HCC cell growth and invasion in vitro. Although these findings have provided some clues to understanding the function of DLGAP5 in HCC, we must further explore the underlying mechanisms and the gene targets of DLGAP5.

## Supporting Information

Figure S1 **Screen of high efficiency RNAi fragment for knockdown of DLGAP5.** (A and B) siRNA1, siRNA2 and siRNA3 were used to knock down DLGAP5 in SMMC-7721 (A) and HepG2 (B) cells, as demonstrated by RT-PCR. siRNA-NC was used as a control.
(TIF)

Figure S2 **Effect of DLGAP5 on HCC cell migration.** (A and B) Migration of SMMC-7721 (A) and HepG2 (B) cells that were transfected with siRNA2 in a wound-healing experiment, with siRNA-NC serving as a control.
(TIF)
