# Supplementary figures and images for: Silencing of DLGAP5 by siRNA Significantly Inhibits the Proliferation and Invasion of Hepatocellular Carcinoma Cells
Source: PLoS One. 2013 Dec 4;8(12):e80789. doi: 10.1371/journal.pone.0080789 (PMC3851768; doi:10.1371/journal.pone.0080789)

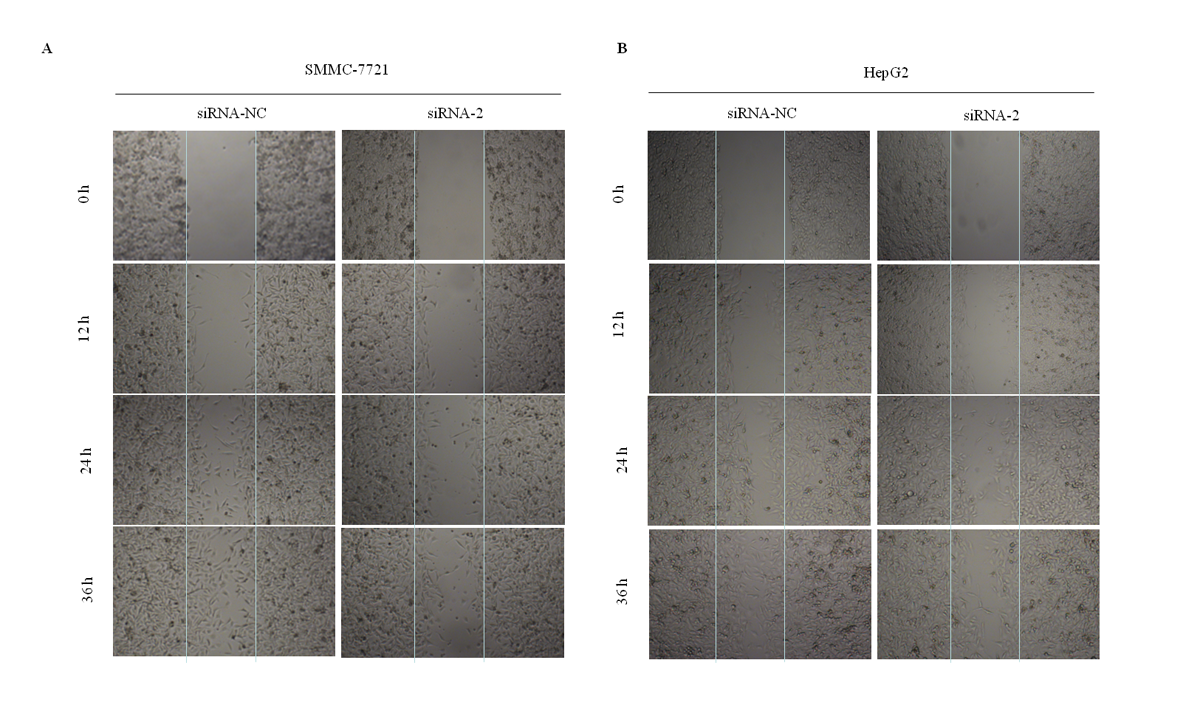

Supplement: Figure S2 — Effect of DLGAP5 on HCC cell migration. (A and B) Migration of SMMC-7721 (A) and HepG2 (B) cells that were transfected with siRNA2 in a wound-healing experiment, with siRNA-NC serving as a control. (TIF) [file pone.0080789.s002.tif]
